# Multifunctional microfluidic chip for cancer diagnosis and treatment

**DOI:** 10.7150/ntno.49614

**Published:** 2021-01-01

**Authors:** Qiao-ru Guo, Ling-ling Zhang, Ji-fang Liu, Zhen Li, Jia-jun Li, Wen-min Zhou, Hui Wang, Jing-quan Li, Da-yu Liu, Xi-yong Yu, Jian-ye Zhang

**Affiliations:** 1 Key Laboratory of Molecular Target & Clinical Pharmacology and the State Key Laboratory of Respiratory Disease, School of Pharmaceutical Sciences & the Fifth Affiliated Hospital, Guangzhou Medical University, Guangzhou, P.R.China.; 2 Department of Gastroenterology, Qilu Hospital, Cheeloo College of Medicine, Shandong University, Jinan, P.R.China.; 3 Guangzhou Institute of Pediatrics/Guangzhou Women and Children's Medical Center, Guangzhou Medical University, Guangzhou, P.R.China.; 4 The First Affiliated Hospital, Hainan Medical University, Haikou, P.R.China.; 5 Department of Laboratory Medicine, Guangzhou First People's Hospital, School of Medicine, South China University of Technology, Guangzhou, P.R.China.

**Keywords:** Microfluidic chip, Cancer, Preclinical model, Drug screening, Biomarker, Nanoparticle

## Abstract

Microfluidic chip is not a chip in the traditional sense. It is technologies that control fluids at the micro level. As a burgeoning biochip, microfluidic chips integrate multiple disciplines, including physiology, pathology, cell biology, biophysics, engineering mechanics, mechanical design, materials science, and so on. The application of microfluidic chip has shown tremendous promise in the field of cancer therapy in the past three decades. Various types of cell and tissue cultures, including 2D cell culture, 3D cell culture and tissue organoid culture could be performed on microfluidic chips. Patient-derived cancer cells and tissues can be cultured on microfluidic chips in a visible, controllable, and high-throughput manner, which greatly advances the process of personalized medicine. Moreover, the functionality of microfluidic chip is greatly expanding due to the customizable nature. In this review, we introduce its application in developing cancer preclinical models, detecting cancer biomarkers, screening anti-cancer drugs, exploring tumor heterogeneity and producing nano-drugs. We highlight the functions and recent development of microfluidic chip to provide references for advancing cancer diagnosis and treatment.

## Introduction

The conventional approach of cancer treatment includes surgery, radiotherapy, chemotherapy, and targeted therapy. These approaches have significant curative effect in the early stage of management, but cancer relapse usually occurs after a period of treatment. Moreover, the “one-size-fits-all” treatment works differently in different patients 1. Every type of cancer needs a unique treatment regimen 2. Therefore, an efficient, rapid and accurate tool is needed to realize the precise diagnosis and treatment for each patient.

Microfluidic chip is an approach which can manipulate fluids on a microscopic scale, thus controlling cell culture relevant parameters to better simulate the microenvironment of tumor tissues *in vivo*. More precisely, the microscale structure of microfluidic chip can delicately operate cells, the multiplexing microstructures are easy to conduct high-throughput analysis, the control of microfluid is advantageous to mimic internal fluidic environment and specific material properties can better mimic the tumor microenvironment. It has a great potential to become a powerful auxiliary equipment to realize in precision medicine 3, particularly in the fields of tumor organoid culture, screening of anticancer drugs, detection of cancer biomarkers, single-cell sequencing and preparation of nanoparticles (NPs). In light of these functions, microfluidic chip combined with downstream analysis can identify molecular, cellular and biophysical features of cancer progression 4. Due to its customizable characteristics, microfluidic devices can meet the needs of various researches, thus has tremendous prospect for development. In this manuscript, we will highlight the multi-function of microfluidic chip and summarize the new technologies to develop novel microfluidic chips.

## Functional diversity of microfluidic chip

### The establishment of preclinical models

Preclinical models are needed for exploring the key molecular and cellular mechanisms of cancers. Microfluidic chip cancer models are often used in testing the efficacy and assessing the safety of the potential anticancer agents or drug combination regimens. Microfluidic devices can not only automate the culture of tumor cells, but also realize multi-cell co-culture under the biomimetic condition by controlling fluid flow rate and other parameters to form cancer tissue organoids. Therefore, microfluidic chips have great potentials in the establishment of preclinical models.

#### Types of preclinical cancer models

Conventional two-dimensional (2D) cancer cell cultures are convenient, but they cannot reflect the complex information of tumor microenvironment (TME) 5. Compared with 2D cell culture, the three-dimensional (3D) cancer cell culture, including 3D hydrogel and 3D spheroids, can better mimic TME, especially the 3D spheroid, which exhibits the complex cellular heterogeneity and the physiologically relevant cell-cell and cell-extracellular matrix (ECM) interactions 6. However, 3D hydrogel cell culture lacks tissue-tissue interface and require large amount of cells. Tumor spheroids in the traditional sense are still unable to reproduce the mechanical forces, such as fluid shear stress which tumor tissues are subjected to 4. Using microfluidic devices to preform 3D cancer cell culture could solve this problem 7-12.

Animal models still play important roles in preclinical trials. The development of therapeutic strategies cannot advance without the results of animal studies 13. However, establishing a proper animal model is very costly and time consuming. Nevertheless, animal models usually lack the native human tissue-microenvironment. Some kinds of animal models, such as nude mouse, are also lack of immune response. Indeed, trials in animal models cannot usually correctly predict the future responses of drug therapy in human 14.

Due to the disadvantage of these preclinical models, tumor organoids models have attracted more and more attention. Organoids is a kind of 3D cell culture that generated from stem cells or organ progenitors, including human pluripotent stem cells (PSCs) 15-17 and cancer stem cells 18-20. Organoids consist of multiple organ-specific cell types and are able to recapitulate some specific function of the organ 21. Tumor organoids derived from cancer stem cells and other cells existing in TME can mimic tumor characteristics *in vitro*, as well as the heterogeneity in tumor. Taken together, tumor organoids have enormous potential in cancer modeling.

**Figure 1:**
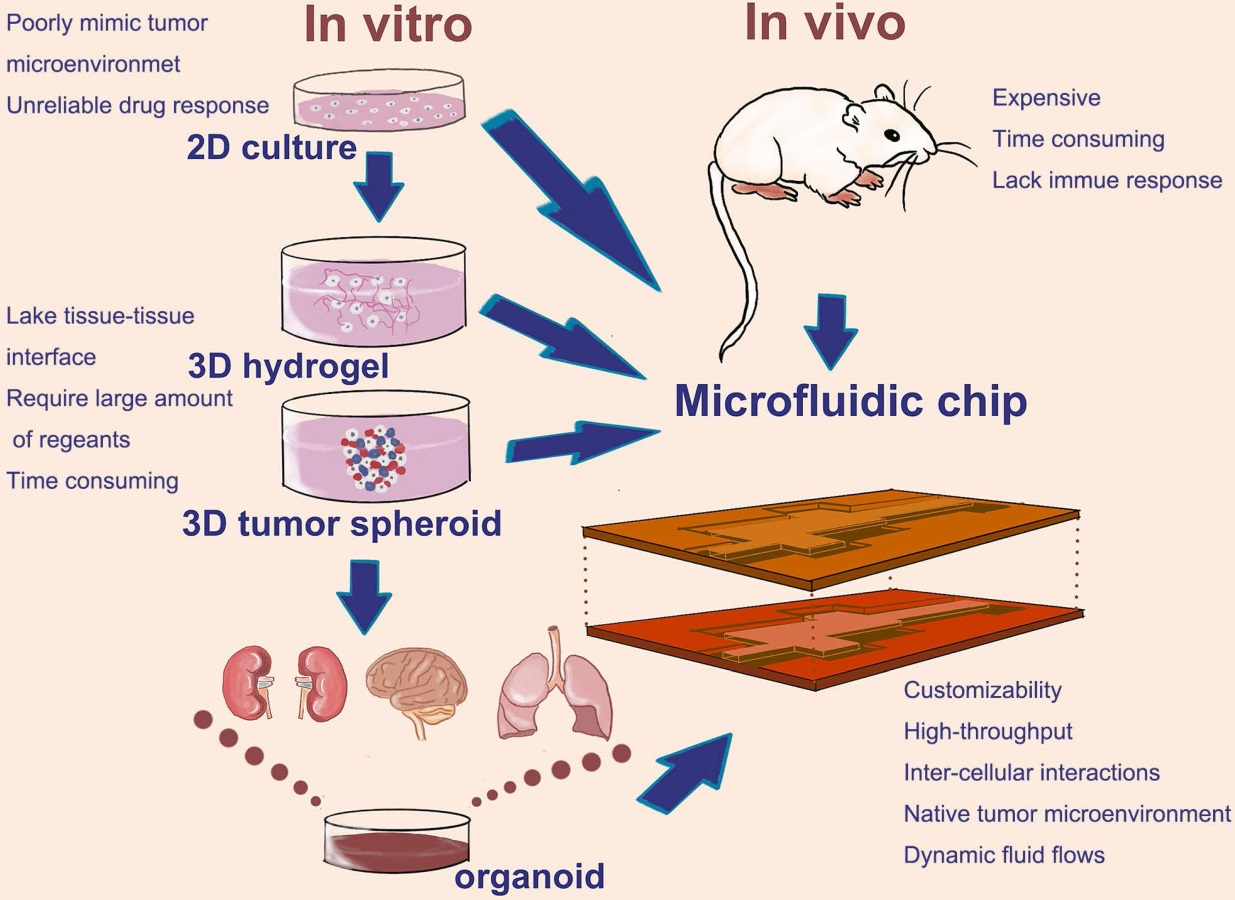
Common types and the development of cancer preclinical model; Animal models, 2D culture, 3D culture, as well as tumor organoid which development in recent years, have playing an important role in cancer preclinical modeling. Microfluidic chip as a promising technology can flexible integrin these cell culture modes on a chip.

Cell culture and cancer modeling at the 2D or 3D level can easily be realized in microfluidic chip. Using the microfluidic chip, the 2D culture, 3D hydrogel, 3D tumor spheroid, as well as tumor organoids could provide reliable data in a high-throughput and automated way. Therefore, microfluidic chip makes it more widespread application in the development of preclinical cancer model (**Figure 1**). How does microfluidic chip mimic tumor microenvironment and establish cancer models? Thus, we will introduce a few examples in the next part.

#### Cancer models on microfluidic chip

The secondary tumors formed through metastasis are the main causes of cancer mortality. Tumor progression and metastasis is a stepwise cascade of events that include the primary tumor growth, angiogenesis, tumor cells invasion, intravasation, extravasation and metastasis to secondary sites 22, 23. Therefore, it is a great challenge to set up an appropriate cancer model on microfluidic chips.

In the past decades, many studies have shown several types of human organ chip models. Hassel et al. reported an orthotopic cancer organ chip model of non-small-cell lung cancer (NSCLC) to recapitulate tumor growth, dormancy and the therapeutic response to tyrosine kinase inhibitors (TKIs) associated with breathing motions 24.

**Figure 2:**
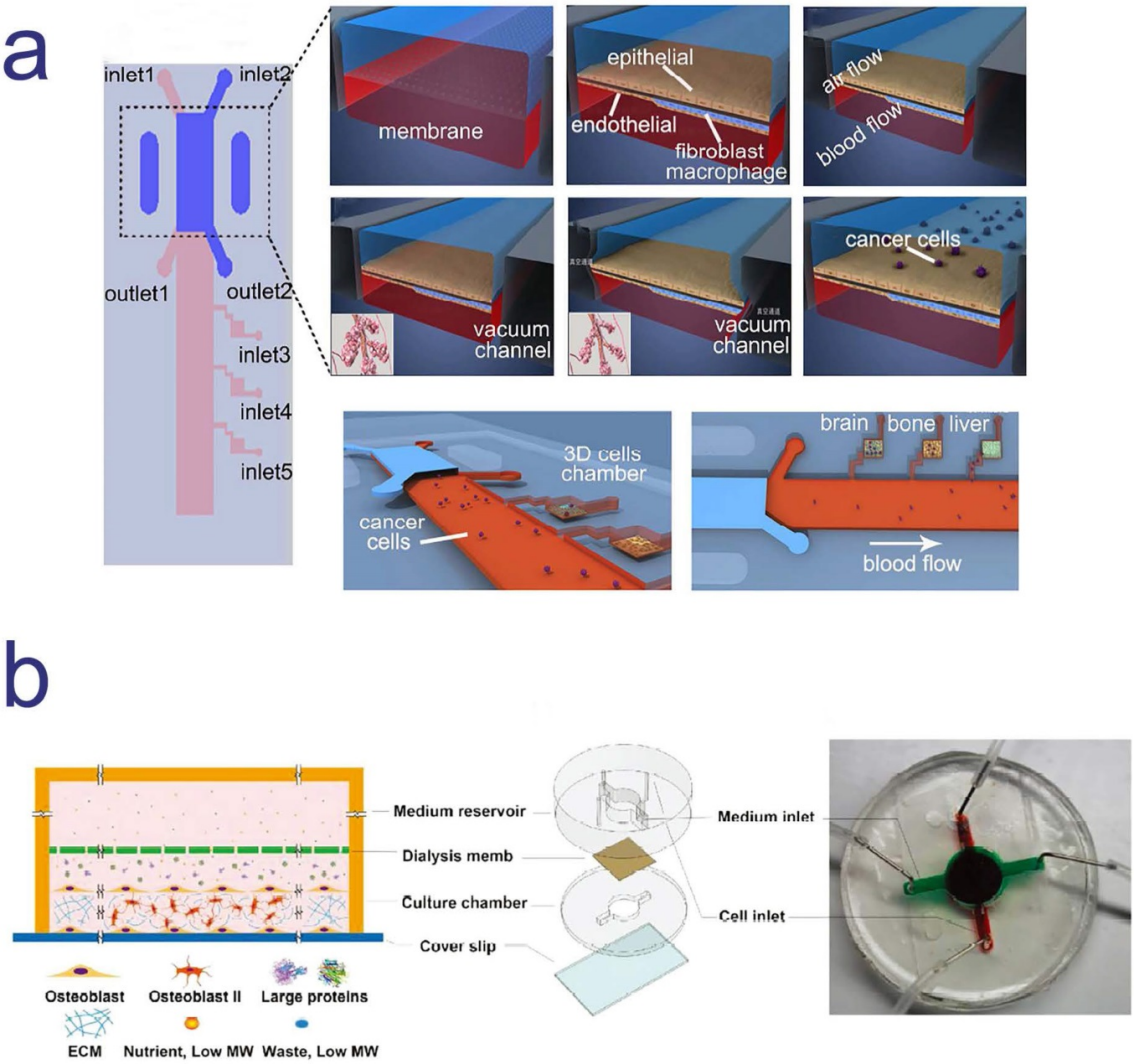
Two different cancer models based on microfluidic chips to mimic cancer metastasis; **a.** a multi-organ microfluidic chip to mimic lung cancer metastasis to the brain, bone and liver; **b.** a cancer model based on microfluidic chip for the study breast cancer metastasized to bone marrow. **a.** Copyright American Chemical Society, 2016. Reproduced with permission from reference 25; **b.** Copyright Wiley, 2018. Reproduced with permission from reference 26.

Microfluidic devices which used to mimic cancer metastasis process are usually applied to several cell types in order to culture two or more organoids. Different organoids are separated by some specific biomaterials, such as polydimethylsiloxane (PDMS), and connected with each other by channels and controllable fluids. Xu et al. designed and constructed a multi-organ microfluidic chip to mimic lung cancer metastasis to the brain, bone and liver. In this platform, organoids were divided into different chambers, including upstream lung organoid and three downstream organoids. Different types of cells were seeded in each chamber to culture different organoids and each organoid were linked by side channels. The culture medium flowed through microvascular channels to simulate blood circulation. At the same time, a circulating vacuum was applied to mimic the physiological breathing (**Figure 2a**) 25. This system provided a physiologically relevant context to recapitulate the complex process of lung cancer metastasis and help us to effectively explore the underlying mechanism of lung cancer metastasis.

In another research, a microfluidic bone chip was used to study breast cancer metastasized to bone marrow. Based on the principle of the simultaneous growth dialysis, the space of bone-on-a-chip (BC) contained two areas for osteoblastic tissue growth and culture medium flow (**Figure 2b**) 26. This design mimicked a natural bone microenvironment and allowed mineralized osteoblastic tissue to form an unprecedented thick layer without artificial scaffolds or *in vivo* growth step. Furthermore, researchers co-cultured the metastatic human breast cancer cells with the osteoblastic tissue developed in the BC and observed several important features of bone metastasis in breast cancer. The BC has the potential to become a powerful tool in the study of cancer bone metastasis* in vitro*.

In addition to the cancer metastasis model, microfluidic devices can be designed into a variety of preclinical models to cater for the needs of various studies, including the microfluidic chip of human 3D microvasculature assay to study cancer cell extravasation 27, 28, a microfluidic platform to study the metastatic cancer cell matrix invasion 29 and the microfluidic blood-tumor barrier model 30. The microfluidic chip models used to simulate the different stages of tumor progression to metastasis are summarized in **Table 1.**

Of course, cancer models are not simply made to replicate the physiological and pathological status. They played a much more profound role in screening of anticancer drugs and the detection and discovery of biomarkers. Large-scale production of reliable microfluidic tumor organoid chip models can achieve rapid and high-throughput drug screening and real-time dynamic monitoring of disease signals in a visual and quantitative way.

#### 3D printed microfluidic chip and cancer models

The integration between 3D bioprinting and microfluidic chip has given microfluidic chip greater potential to model cancers. Traditionally, in cancer modeling on chip, microfabrication such as micromachining, photolithography and injection molding, are used in the fabrication of microfluidic chips 31. These methods have high resolution and accuracy, but their high cost, complex process and difficult reproducibility greatly limited the development of microfluidic chip 3. The emerging of 3D printing technology greatly simplifies the fabrication process of microfluidic chips.

In addition to manufacture microfluidic device, 3D printing technology can be applied to construct organ-on-a-chip and biological scaffolds on chips 32, 33. Four common methods of 3D printing used in bioprinting and chip fabrication including stereolithography (SLA) bioprinting, extrusion bioprinting, inkjet bioprinting and laser-assisted bioprinting. The application of 3D printing provides a new method for cell seeding on microfluidic chips without the need for the time-consuming manual seeding and the redundancy pumping seeding 34. Not only that, 3D printing allows the simultaneous process of living cells and biomaterials, which provides tumor model with fine, replicating ECM on chips 35. Using specific bio-ink formulations, 3D bioprinting is able to build different complex channels or ECM on chip and preserve the heterogeneity of the primary tumor 34, 36. In fact, for the organoid or biological scaffolds constructed by bio-printer, applying the mechanical force generated by fluid is able to simulate the metastasis of cancer cells *in vivo*. It has been reported that cancer cells tend to migrate towards the direction of liquid flow, and the microfluidic chip provides a mechanical force to mimic TME 37. These merits allow 3D printing to enhance the function of microfluidic chips, and 3D printing gradually became an important method in cancer modeling and drug screening on chips 38-41. Several representative studies on the use of 3D printed microfluidic device for cancer modeling or diagnosis in the past five years was summarized in **Table 2.**

### The detection of cancer biomarkers

Cancer biomarkers could be circulating tumor cells (CTCs), circulating tumor DNA (ctDNA), exosomes, non-coding RNA (ncRNA) and various cellular metabolites or proteins 42-44, of which the precise detection of biomarkers contributed to early diagnosis and grading of cancers 45. Conventional cancer screening methods, such as invasive tissue biopsy or medical imaging, are costly and complex. Recently, microfluidic chip is catching up to overcome these obstacles.

#### On-chip CTC detection

CTCs are tumor cells that shed from solid tumors into peripheral blood. Several studies indicated that CTCs are closely associated with cancer metastasis. The significance of using CTCs as cancer biomarker is that CTCs have the ability to reflect the real-time tumor burden and explore tumor heterogeneity. However, detecting CTCs from blood is extremely challenging for three reasons. First, CTCs are so rare: it could only be one CTC per 10^9^ blood cells in the patients' peripheral blood 46. Therefore, it might require a large quality of blood samples. It is extremely low limit of detection (LOD) to draw CTCs from blood samples. Second, CTCs varies in size and morphology, making it difficult to identify. Third, CTCs are easily damaged in the process of identification 42.

The existing gold standard for CTCs separating and counting is CellSearch© system, which is based on immunoaffinity for isolation and fluorescence for cell counting 47. In this system, epithelial cell adhesion molecule (EpCAM) as a selection tag for CTCs, anti-EpCAM conjugated magnetic beads are used to capture and isolate CTCs 48. In fact, immuno-magnetic capture is a very classical method and EpCAM is a common antigen in CTCs capturing. In addition to CellSearch©, immune isolation is used in most CTCs separation platform, such as MagSweeper 49 and many microfluidic CTCs isolation platform.

However, the antigen-based selection of CTCs has some drawbacks. First, it has been shown in some studies that cell metabolic and protein composition may alter because of the use of antibody against EpCAM 50. Second, antibody binding to cell surface antigens may cause cytotoxic effects 51. Third, the number of CTCs is sometimes underestimated due to antigenic bias. Moreover, both EpCAM-positive or EpCAM-negative CTCs are circulating in blood 52. Therefore, besides the antigen-based selection, based on some physical properties, such as the size 53, 54, shape 55, electrical impedance 56 or inertial focusing of cells, these have also proved to be effective. In addition to these methods, Ganesh et al. proposed another approach for isolation of CTCs. According to the Warburg effect, glycolysis is upregulated in primary and metastatic cancers 57. Glucose is metabolized into pyruvate and lactate, leading to the low pH in extracellular environment of cancer cells compared to normal cells 58. Researchers used the pH differences between normal cells and cancer cells to set up a microfluidic chip based on ZnO pH sensors for possible identification of CTCs in blood 59. Even the antigen-independent method still have some drawbacks (for example, some studies have shown that CTCs are similar or smaller in size to leukocytes; the effect of hydrogen, oxygen and elevated temperatures generated by the use of dielectrophoretic method cannot be ignored), it still can cover some shortage of the antigen-based selection of CTCs to some extent.

Nowadays, more and more studies have applied not only a single CTCs capturing method, but also a hybrid method combining these two classical modes, among which CTC-iChip is a representative CTCs isolation platform. The main design principle of CTC-iChip is the high-efficiency negative depletion of blood cells. At the same time, CTC-iChip utilized the inertial forcing to align nucleated cells, deflect and collect magnetically tagged cells. The untagged CTCs isolated from CTC-iChip are easy to purify the high-quality RNA, which is particularly suitable for downstream transcriptomic analysis 60. Yan and colleagues developed a “Rhipsalis (Cactaceae)”-like Hierarchical Structure on chip. This platform combined two approaches based on cell size or immunoaffinity to capture CTCs by modifying specific antibody on the micropillars 61. Both methods have high capture efficiency and are wildly used in many microfluidic devices. The downside is the high production cost and time-consuming.

In order to achieve more efficient CTCs capture and isolation, more and more studies tend to combine traditional techniques (such as immune-magnetic capturing) with microfluidic chip or image processing during these years 62. It is worth to mention that microfluidic shows great application potential in CTCs capturing due to its multiplexing and simplicity. Various CTCs isolation platforms based on microfluidic chips have appeared one after another, such as CTC-chip, CTC-ichip, spiral chip, GEDI and so on. In **Table 3**, we summarize several representative CTCs isolation microfluidic chip in the past decades.

Microfluidic chip is usually used for isolating and enumerating CTCs. However, with the in-depth study on CTCs, scientists gradually found that besides evaluating the number of CTCs in a certain volume of blood to determine tumor burden, CTCs also play a key role in cancer metastasis and reflect the tumor heterogeneity. For this reason, after isolating CTCs, the downstream analysis of CTCs can better provide molecular characteristic. For example, protein analysis and single cell sequencing can help to increase the recognition of cancer subpopulation and provide a powerful reference for personalized therapy. Microfluidic Western blotting has been reported to profiling protein expression in patient-derived single CTCs 63. In another study, combining with multiplex surface-enhanced Raman spectroscopy (SERS) nanovectors on microfluidic chip was used to identify subtypes of CTCs in accordance with the clinically relevant surface protein composite spectral signatures 64. More remarkably, using single-cell sequencing on CTCs or other cancer relevant cells on chips have been wildly used, which will be elaborated in the section 2.4.

#### On-chip exosome detection

Exosome is one of the extracellular vesicles (EV) and the size ranges between 30-100 nm 65. Exosomes act as communicators between different cells by transferring a variety of cargoes, such as mRNA, ncRNA and proteins 66. In recent years, exosome has been considered as a promising biomarker for cancer diagnosis and prognosis. Changes in the expression of certain cargoes in exosome tend to suggest tumor status or indicate some changes taken place in tumor.

Cancer derived exosomes are often extracted from various body fluids, such as serum, ascites, and pleural effusion 67. The most commonly used exosome separation method is to use ultracentrifugation (UC) 68, which is time- and reagents-consuming. Although various new methods emerged in recent years, there is not much change in exosome separation and detection 69-71. Microfluidic chip can not only efficiently complete the separation and detection of exosomes, but also integrate these two techniques on a single chip, which greatly simplify the procedure.

Microfluidic chip is like a framework that could be flexibly used by researchers based on their study needs. There have been various methods to separate and detect tumor derived exosomes on chips. The separation methods include immunoaffinity-based separation 72-74, nanomembranes filter 75, dielectrophoretic (DEP) separation 76, lateral displacement and acoustic fluid separation 77. The detection techniques include fluorescence detection, electrochemical detection 78 and mass spectrometry 79. Moreover, there are integrated chips that combined exosome separation and detection in the last few years. For example, Xu et al. set up a two-stage microfluidic platform which integrated a staggered Y-shaped micropillars and an Indium Tin Oxide (ITO) electrode 80. This platform achieved separation of exosomes separation by the newly staggered Y-shaped micropillars array to create anisotropic flow and promote the full binding of exosomes to antibody modified magnetic beads. Following capture, using a cascading ITO electrode to detect the captured exosomes and realize signal transduction 80.

The main challenges of the exosomes isolation are to achieve high-throughput, high recovery rate and low damage 81 for rapid detection. Although great achievement has been made in the field of exosome detection on chips in the past years, there is still much space for improvement in the existing techniques.

#### On-chip ctDNA and ncRNA detection

Circulating nucleic acids are released from the apoptotic cancer cells or tumor exosomes that entered blood circulation 82. The levels of circulating nucleic acids reflect the tumor burden or malignant progression 83. Circulating nucleic acids including cell-free DNA (cfDNA), mRNA and ncRNA, et al. ctDNA is a sub-class of cfDNA which carries information of mutations and often detected in the peripheral blood of cancer patients 84. ncRNA is a kind of RNA that has no coding function, including microRNA (miRNA), long noncoding RNA (lncRNA), circular RNA (circRNA), et al. Many studies have shown that ncRNAs are functional regulatory molecules that involved in cancer progression 85-88.

The equipment used for extracting ctDNA through liquid biopsy needs to meet the ctDNA size ranged 50-150 bp and with high recovery, minimal interference, and high reproducibility 89. Microfluidic chip provides an automated platform that greatly improved the efficiency of ctDNA extraction. The solid-phase extraction technique was applied to a microfluidic chip to activate the polymer surface to generate -COOH by UV/O3. The cfDNA was extracted from patients' plasma samples through the specific immobilization buffer, achieving the purpose of high recovery and low cost 90.

Furthermore, point-of-care (POC) cancer diagnostic is one of the goals in the development of microfluidic chip. Since the half-life of cfDNA are very short, the rapid and automatic techniques used to isolate cfDNA from plasma with low degradation are urgently needed. Kim and colleagues developed a fully automated microfluidic platform that can purify cfDNA from cancer patient`s plasma in a short time (30 min). This device is based on the electromagnetically actuated diaphragm valves. It integrated the function of plasma separation, residual protein lysis, cfDNA elution, therefore greatly improved the time of cfDNA isolation from patients' blood.

Many studies have demonstrated the tremendous application of using the combination of microfluidic chip and digital polymerase chain reaction (PCR). The reason is that compared to the commercial PCR assay, digital PCR technique on chips is more accurate, high throughput and less time-consuming 91-94. Digital PCR on microfluidic chips could be used to directly investigate the association among cancer ncRNA 95 and DNA methylation 96 from liquid biopsy. For example, Moltzahn et al. developed a microfluidic platform with multiplex qRT-PCR to profile miRNA signature in the serum of patients with prostate cancer for diagnosis and prognosis 97. Wang et al. used droplet digital PCR (ddPCR) and achieved lung cancer related miRNA qualification 95.

PCR technology is a gold standard for miRNA measurement, but there are some limitations, including the design of suitable primers and the error-prone amplification steps. Recently, the use of the clustered regularly interspaced short palindromic repeats (CRISPR)-associated methods towards the detection of nucleic acid have emerged 98, 99. By using different CRISPR-associated (Cas) effectors, different types of nucleic acid can be detected. Cas13a is an RNA-guided RNase which can produce multiple cleavage sites in nontarget single-stranded RNAs 100, 101. Based on this characteristic, Cas13a can be used to amplify nucleic acid signals without the synthetic nucleic acid amplification steps 102. On microfluidic chips, the enzyme Cas13a, the target-specific CRISPR RNA (crRNA) and the labeled reported RNA (reRNA) combined with an electrochemical biosensor was used to detect miRNA in the serum samples of patients who suffered from brain tumors 103. When the serum sample contained target miRNA, the Cas13a/ crRNA complex caused reRNA collateral cleavage due to the “collateral activity”. The change of reRNA was detected by the electrochemical biosensors and the current signal readout is inversely proportional to the concentration of miRNA in the serum sample 103. It is worth mentioning that the detection time of this method is short (less than 4 h) and the needed sample volume is less than 0.6 μL. All in all, the combination of microfluidic device and CRISPR technique provides a new idea for the detection of circulating nucleic acids and holds promise for the future of POC testing and personalized therapy.

### Anti-cancer drug screening and nano-drug preparation

#### Anti-cancer drug screening on microfluidic chip

According to the culture modes, the microfluidic models for drug screening are categorized as single cell line culture, multi-cell line culture to mimic TME and patient-derived tumor organoid. In addition to evaluating the efficacy, chemosensitivity and safety of a single drug, microfluidic chip can also provide patients with a reasonable drug combination regimen according to their own conditions. These functions allow the determination of specific types of drugs in advance for possible emergence of drug resistance.

##### Drug screening on microfluidic cancer models

**Figure 4:**
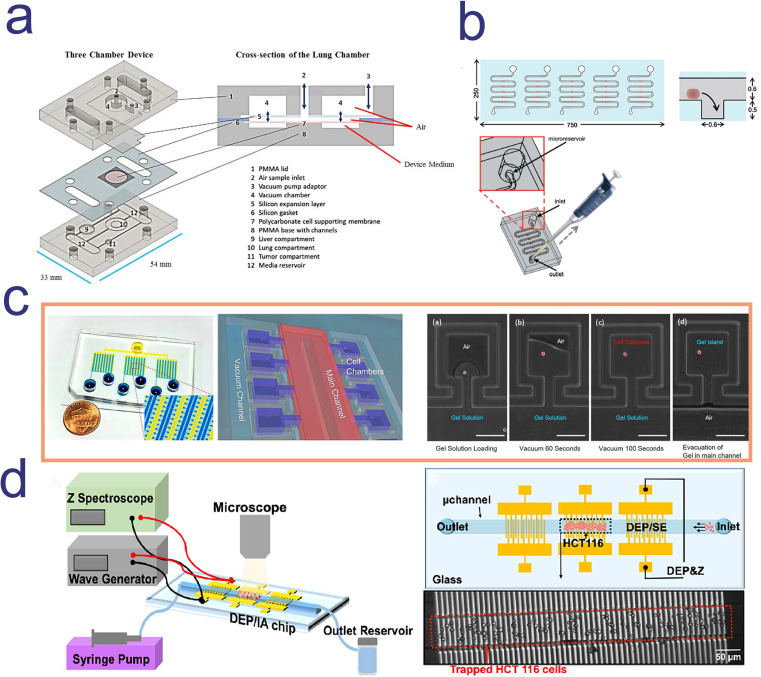
Four microfluidic chip for anti-cancer drug screening; **a.** a three-chamber microfluidic chip consists with a breathable lung compartment, which can better mimic lung breathing mechanisms and easily compare the cytotoxic effects of drug administered by intravenous injection and inhalation; **b.** MDT was trapped by sedimentation in square-bottom wells to avoid excessive shear stress and obtain more stable imaging observation; **c.** Each island of this microfluidic device was formed by gel that pumping out of the main channel and single cells were loaded into each island and maintained high viability; **d.** dielectrophoresis (DEP)/impedance analysis (IA) chip was consists in this microfluidic platform, which can realize high-throughput single cell capture. **a.** Copyright Wiley, 2020. Reproduced with permission from reference 105; **b.** Copyright The Royal Society of Chemistry, 2015. Reproduced with permission from reference 106; **c.** Copyright The Royal Society of Chemistry, 2016. Reproduced with permission from reference 117; **d.** Copyright American Chemical Society, 2019. Reproduced with permission from reference 115.

Setting up a microphysiological system (Body-on-a-Chip) is one of the ways to screen drugs and determine the mode of administration. Inhalation therapy is an important treatment for lung diseases, in which drugs are inhaled directly to the desired sites with less drug accumulation at nontargeted sites 104. A microfluidic platform with multi-organ and breathable lung chamber was reported for the screening and development of inhaled and intravenous drugs 105. In this model, the lung compartment was linked with the liver and tumor compartment by channels. It is worth mentioning that researchers modified the traditional lung air-liquid interface (ALI) model and designed an “ALI bridge” to mimic lung breathing mechanisms (**Figure 4a**). Through the “ALI bridge”, the platform can confirm whether inhaled therapeutic drugs can be used for treating systemic disease. In addition, researchers used the improved hanging drop method to introduce several types of cells and create 3D structure of breast-cancer tumor 105. Based on this platform, researchers can easily compare the cytotoxic effects of curcumin administered by intravenous injection and inhalation.

Utilizing patient derived tumor tissue for microfluidic-based chemo-sensitivity assay has become an important means for personalized therapy. Astolfi and colleagues described a method, named micro-dissected tissues (MDTs), in which patient derived tumor tissues were sectioned to submillimeter size 106. MDTs were trapped by sedimentation in square-bottom wells, because trapping cells by sedimentation can shield MDTs from excessive shear stress and provide more stable environment for imaging and observation (**Figure 4b**). A high-grade serous ovarian cancer patient tissue sample was used to conducting drug screening on the chip. Compared with the clinical follow-up, it was found that the positive response measured by microfluidic chip *in vitro* was consistent with the clinical response of patient, indicating that the platform can identify potential responder 106.

In fact, the generation of quiescent microvascular networks always precedes the nascent tumors during tumorigenesis 107, 108, However, some studies showed that excessive tumor growth and insufficient vascular growth occurred when endothelial cells and tumor cells were seeded at the same time. By adjusting the seeding order of tumor and endothelial cells, Shirure et al. developed a patient-derived organoid microfluidic platform that can simultaneously test chemotherapeutics (such as paclitaxel) and anti-angiogenics (such as bevacizumab). After 7 days culture, the microvascular network was mature and patient-derived organoids were transplanted to the vicinity of the microvascular network, which reproduced the intravasation of tumor cells 109. Moreover, through the microvascular networks, drug testing based on this platform better replicated the physiological delivery of drugs to tumor.

##### Drug screening in single-cell analysis

Anti-cancer drug screening by bionic microfluidic chip is often limited by the collective cell behaviors. Due to the hallmark of heterogeneity in tumor, various cell sub-populations exist in tumors, and some of them are the key factor for cancer metastasis, drug resistance and tumor relapse. Analysis focusing on each individual cell is increasingly important.

Considerable evidence suggests that microfluidic chip has become a state-of-the-art drug screening approach in the single-cell level. A variety of methods based on microfluidic devices have been developed for flexible use in the single-cell manipulation, such as: optical tweezers 110, droplets 111, magnetic beads 112, and deterministic lateral displacement (DLD) separation method 113. Identifying tumor cells by electrical sensing modality (such as measuring cell impedance magnitude) 114, 115, Raman or fluorescence spectroscopy 116 and polymerase chain reaction (PCR) were developed.

For example, a microfluidics 3D gel-island chip was reported to isolate single cell, categorize the cancer cell state and detect single cell drug susceptibility. 3D gel-island was a 3D ECM cell culture environment and each island were formed by gel that pumping out of the main channel. Single cells were loaded into each island and maintained high viability (**Figure 4c**) 117. Utilizing this device, researchers monitored the drug resistant behavior of cells with single cell resolution after treating doxorubicin and cisplatin. After the administration, breast cancer stem-like cells and non-stem-like cells shows different drug resistant behavior, in which stem-like cells were more resistant than non-stem-like cells 117. This result indicated that drug sensitivity was correlated with the change of status of cells and confirmed the great potential of using microfluidic single cell analysis platform for anti-cancer drug screening.

Drug testing methods often require high sensitivity in screening drugs in specific cell population and monitor cell status in limited patients` tumor tissue sample or blood 118. Aside from the costly label reagents, the expensive optical equipment and complex microfabricated channel structures, a new microfluidic device using patient biopsies for drug screening has attracted attentions. The biggest characteristic of this platform is the label-free capture and analysis of targeted cells in real-time. Using the powerful dielectrophoresis (DEP) technique, high-throughput cell capture can be simply performed (**Figure 4d**). In addition, real-time and continuous cellular behavior analysis generated thousands of data point for each therapeutic-cell interaction 115.

#### The preparation of nano-drugs

Some chemotherapeutic and imaging agents with low molecular weight cannot be retained effectively in blood and tumor. NPs are an excellent tool to attack the targeted cancer cells while retain in healthy tissues. The enhanced permeability and retention (EPR) effect allows solid tumors selectively accumulate NPs 119. Small size NPs can passively accumulate in tumors according to EPR effect and can also actively bound to target cells by surface target ligand modification 120. Nanomaterial encapsulation of drugs can reduce toxicity and achieve drug tolerance, while encapsulated imaging agents or modify fluorescent probe are contributed to diagnostics and biological distribution 121.

Compared with the classical batch technology, the microfluidic process are particularly appealing in the synthesis of nanomaterials and the preparation of nano-drugs 122. Micromixer integrated with microfluidic chip provides an efficient mixture in a small length scale; the precise control of temperature and kinetics ensure the uniformity of nano-drugs. In addition, microfluidic device can also satisfy the *in-situ* monitoring of NPs formation. For example, adjusting the flow rate ratio and different lipid components can precisely controlled the NPs size and surface properties 123. Microfluidic devices are now capable of preparing a variety of NPs, including lipid-based nanobiomaterials 124-126, polymeric nanoparticles 127-129, lipid-polymer hybrid nanoparticles 130, 131 and engineered exosomes 132, 133.

Since the flow state of the fluid on microfluidic device is different from that of the turbulence in large-scale channels, its laminar flow state and mass transfer is completely dependent on diffusion 134. Therefore, the mixing step on chip often needs external mechanism, such as electrokinetic 135, 136, magnetic 137, 138 and the special design of channel geometry mentioned above 139, and often lacks the dynamic control of fluid interface. Under this premise, the combination of hydrodynamic focusing (HF) device and microfluidic chip can be a good choice for the synthesis of NPs. In simple terms, the HF process is a high flow rate sheath fluid compresses a low flow rate central fluid 140. In practice, the precise control of relative flow rate of chemical components can regulate the concentration and solubility 129, thus the synthesis of NPs in microfluidic hydrodynamic flow focusing (HFF) device will produce a more uniform particle size distribution. Ran et al. developed an HFF platform for single-step preparation of multifunctional liposomes. In this platform, the plain liposomes, PEGylated liposomes and the folic acid modified liposomes that encapsulated fluorescence dye were synthesized and showed reliable stability in serum. The liposomes modified targeting ligand (folic acid) demonstrated stronger selectivity and internalization in 3D tumor spheroid model 141. Conventional production of multifunctional liposomes often requires tedious post-processing, but this platform greatly reduced the difficulty of liposomal preparation and increased the uniformity of liposomes. More than that, a number of studies in recent years had proven that this technique has tremendous potential for high-throughput production of NPs 142, 143.

Molecular engineering of exosome is a new avenue for drug delivery. In fact, endogenous drug delivery system (DDS) often outperforms synthetic nanomaterials in terms of retention time and targeting. Red blood cell membrane, white blood cell membrane, cancer cell membrane (CCM) and other natural cell membranes have a better biocompatibility *in vivo* and are good raw materials for NPs synthesis 144-147. It is worth mentioning that exosome membrane (EM) can also be used to prepare NPs after engineering. Conventional microfluidic devices have low efficiency in preparation of nanoparticle of natural membrane sources. Liu and colleagues applied microfluidic sonication to assemble tumor-derived EM-coated and CCM-coated poly (lactic-co-glycolic acid) (PLGA) NPs. EM- and CCM- coated NPs have the ability to enhance targeting efficacy because there are some specific surface antigens on their membrane. They can be modified to improve tumor targeting or reduce the clearance of NPs by mononuclear phagocyte system (MPS) 148. This study showed that tumor-derived EM-coated NPs have better homotypic targeting. The underlying mechanism of this phenomenon might be that tumor cell-derived EM have both the endosomal and plasma membrane protein, which makes EM-coated NPs have the dual function of avoiding immune clearance and targeting homologous tumors 149. Although there have been limited reports on the use of microfluidic device for exosome engineering, due to the unique properties of exosome membranes and the flexible functions of microfluidic devices, this research field will have a great prospect in the future.

Using microfluidic device for preclinical evaluation of NPs has also shown advantages. The special design of channel geometry (such as line and cross shape microstructure) and highly controlled fluidics provides a high fluid mixing and avoids pure laminar flow and NPs sedimentation, thus increasing the internalization of NPs by the cells^139^. Because of the enhanced permeability and retention (EFR) effect, NPs often accumulate in tumors. The technical Tumor-Vasculature-on-a-Chip (TVOC) was reported to assessing NPs extravasation through leaky vasculature and their accumulation in tumor tissues, which provided a powerful platform for the preclinical evaluation on NPs 150. Chen and colleagues recently developed a microfluidic platform that composed of the breast-cancer multicellular tumor spheroids (MCT) with uniform size, the endothelial monolayer and ECM, which better recapitulated the pathophysiological barrier and the blood microvessels in breast cancer microenvironment 151. More than that, real-time monitoring on the chip through microplate reader is more accurate than conventional fluorescence detection 152-154. On the strength of these features, the researchers synthesized a carbon dots (CDs) drug delivery system as a model to monitored drug delivery capacity and assessed *in-situ* cytotoxicity on the chip 151. The results showed that this microfluidic platform give the possibilities of integrating useful characteristics of high-throughput and high spatio-temporal resolution in nano-drug evaluation.

### Exploring tumor heterogeneity on microfluidic chip

Tumor heterogeneity is an ongoing challenge in cancer therapy, which can be divided into intertumoral heterogeneity and intratumoral heterogeneity. Intertumoral heterogeneity refers to the heterogeneity between different patients' tumors with the same histological type 155. Intratumoral heterogeneity means genomic diversity within a single tumor 156. A single tumor may contain different subclones, such as tumor-infiltrating cells, supportive cells and transformed cancer cells 157. How to identify the heterogeneous clonal landscape of tumor and determine different drug combination regimens in different patients according to the situation of individual patients is a major problem in the realization of personalized therapy.

For single-cell sequencing, the main question we need to explain is that which gene or pathway defined cell status and how cell status affected disease 158. The combination of microfluidic technique and high-throughput sequencing has been proved to be of great value for large scale analysis on single cell transcriptome. Captured and lysed a single cell is the first step on the single-cell sequencing carried out on microfluidic chip. Streets et al. developed an on-chip single-cell whole-transcriptome sequencing. After capturing and lysing cells, the mRNA with ploy A tail was reversely transcribed into cDNA and finally, using the next generation sequencing platform to collect the double-stranded cDNA for single-cell sequencing 159. This model is of great significance and prospect for studying tumor heterogeneity because the detection sensitivity and measurement accuracy of mRNA have been significantly improved after the integration of microfluidic chip.

Targeting the specific oncogenic driver mutation gene can effectively inhibit tumor progression. For non-small cell lung cancer, patients with epidermal growth factor receptor (EGFR) mutation often choose EGFR tyrosine kinase inhibitors (TKIs) in clinical practice 160. Therefore, the identification of EGFR mutations is crucial for targeted therapy 161. Utilizing silicon-designed microwells on microfluidic chip, NSCLC cells was trapped into each microwell and imaging by immunofluorescent. After *in situ* lysing each cell, the cost-effective Sanger`s sequencing was used to find out multiple mutations 162. EGFR-mutated cells make up only a small proportion of the whole cancer cell population. This device has been proven to eliminate the noise of most un-mutated cells and accurately identify the mutation in which cell and determine whether different mutations co-exist in the same cell, providing reference for the clinical TKI selection. Similarly, the combination of the tri-states valve structure and Sanger`s sequencing enabled high-throughput processing of multiple cells. With the integration of single cell capture, identification, lysis and *in situ* DNA MDA (Multiple Displacement Amplification) on a single chip, the statistical information on oncogenic mutations will be provided cost-effectively 163.

Besides single-cell sequencing, it has been reported that the lactate release level can be measured by microfluidic chip for sorting and identifying single cell. The level of lactate released by cancer cells during glycolysis is often related to the tumor metastasis, drug resistance and relapse 164, 165. Mongersun et al. developed a microfluidic platform that utilized droplet to encapsulate a single cell. This platform measured not only extracellular lactate concentration, but also lactate release rate in the single-cell level. Under the chemical inhibition of lactate efflux, researchers identified the malignant cells and explored cancer metabolic pathway by studying the differences in two cancer cell lines 166. Beyond that, droplet microfluidic chip usually needs surfactant to stabilize droplet formation. The droplet interfacial tension of a specific surfactant is sensitive to pH. The relationship between interfacial tension and pH can affect the droplet flow, and thus the droplet contained live cells can be sorted 167. The method of using interfacial tension on microfluidic chip to sort cancer cells in different metabolic status proposed a new idea for single cell analysis.

## Conclusions

The biggest characteristic of microfluidic chip is the customizability, which means microfluidic chip is a very flexible scientific tool that can accommodate with advanced technologies. To date, microfluidic chip shows tremendous promise in cancer diagnosis and treatment. Microfluidic chip can be applied in everything from anticancer drug development and screening to cancer modeling and diagnosis. In recent years, the application of 3D printing in fabrication of microfluidic devices has made expensive photolithography no longer the only way to fabricate microfluidic chips. Because of the customizability and flexibility of microfluidic chip, establishing complicated tumor organoid on microfluidic chip is available, and patient-derived tumor tissue is able to be cultured and analyzed on a tiny chip.

There still exist some challenges on the development of microfluidic chip. The advantage of microfluidic chip is that it is easy to observe cell response and tumor morphology through imaging on microfluidic chips. For example, immunofluorescence is the most common method to analyze cell response on the chips. However, it is a challenge to collect cell samples from chips. In the process of sample collection, a number of chips need to be disassembled, which easily causes contamination of cell culture environment. At the same time, samples could be damaged in the collection process. This disadvantage hinders some experimental operations, such as immunohistochemistry. For this reason, a more reliable sample collection for microfluidic chip is urgently needed to be developed. In addition, PDMS is still the most commonly used material for microfluidic chips due to its excellent performance of biocompatibility, optical transparency, permeability, and the low cost. However, it has been reported that PDMS may exist physical or chemical reactions with certain reagents, and different PDMS formulations may have different interactions with different cells, which may bring trouble in some studies related to cell culture and drug screening. Although some obstacles can be overcome by adding specific coatings to culture region and other skills, developing new materials to fabricate microfluidic chips will bring us more options for scientific research.

In the past three decades, microfluidic chip has been rapidly developed. Although there are challenges need to be overcome, many achievements have been made in the field of cancer diagnosis and treatment. In the new era of personalized medicine, microfluidic chips should be developed toward accurate and point-of-care cancer diagnosis, bringing hope for personalized cancer treatment.

## Figures and Tables

**Figure 3:**
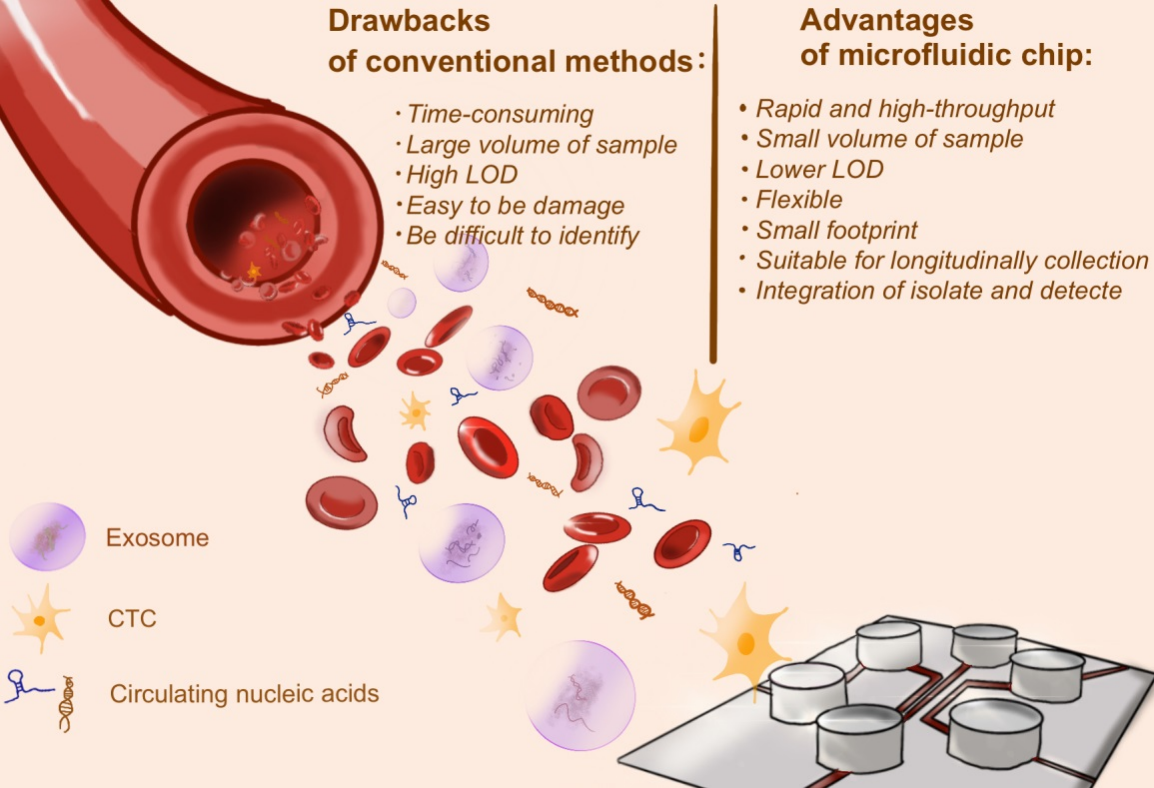
The advantages of microfluidic chip for cancer biomarker detection. Conventional methods to detect cancer biomarkers, including invasive tissue biopsy or medical imaging, are costly and time-consuming, and even need large volume of sample. Microfluidic chip, as a burgeoning approach, shows great potential in biomarkers detection. The merits are described in the figure above.

**Table 1:** List of microfluidic cancer model about mimicking the cascade events of tumor progression

Aim of study	Culture model type	Cancer type	Notes	Ref.
Tumor-like transformation	2D culture	Lung cancer	Studying tumor-like transformation of bronchial epithelial cells that are continuously exposed to cigarette smoke extracts	168
Lesions of ductal carcinoma *in situ*	3D culture	Breast cancer	Tumor spheroid co-cultured with mammary fibroblasts and human mammary ductal epithelial cells to mimic 3D structural organization and microenvironment	169
Angiogenesis	3D culture	-	Using a newly Sphero-IMPACT platform to culture 3D tumor spheroid and monitor angiogenesis, tumor cell migration and invasion	9
Extravasation	3D culture	Breast cancer	Setting up a 3D microvascular network to study human metastatic breast cancer cell extravasation	28
Extravasation	3D culture	-	Establishing a human microcirculation model to dynamically monitor the extravasation of several tumor cell line	27
Extravasation	3D culture	Breast cancer	Establishing a microvascular network to study the extravasation potential of breast cancer cells in a hypoxia environment	170
Invasion	3D culture	Lung cancer	Using composite hydrogel microfibers to quantitatively analyze invasion behavior of tumor cells	171
Invasion	3D culture	Breast cancer	Formed a tumor-macrophage bidirectional crosstalk system to evaluate the antagonistic effect of the system on anticancer drugs	172
Metastatic cancer cell matrix invasion	3D culture	Breast cancer	Cancer cells co-cultured with endothelial to explore the matrix invasion behavior of metastatic breast cancer cells	29
Invasion and migration	3D culture	Breast cancer	Tumor cells co-cultured with patient-derived fibroblast cells and evaluate tumor cell migration and invasion under the influence of tumor-stroma interactions	173
Invasion and migration	3D culture	Breast cancer	Through polyelectrolyte complex coacervation process, 3D collagen barrier was formed around cancer cell to mimic the basement membrane and observe cells migration and invasion	174
Migration	3D culture	Breast cancer	Tumor spheroid co-cultured with endothelial cells. Using 3D photopatterning to confine cells into gelatin methacrylate (GelMA) hydrogel structures	175
Metastasis	3D culture	Breast cancer	Developing a spontaneous “bone-on-a-chip” to study bone metastasis in breast cancer	26
Metastasis	2D culture	Breast cancer	Developing a microfluidic blood-tumor barrier model to study brain metastasis in breast cancer	30
Metastasis	3D culture	Colon cancer	Developing a multiple 3D tissue construction to study liver metastasis in colon cancer	176
Metastasis	3D culture	Colorectal cancer	Using 3D photopatterning technique, researchers developed a microfluidic device that houses lung and liver organoid to mimic lung and liver metastasis in colorectal cancer	177
Stroma-mediated cell motility	3D culture	Pancreatic cancer	Tumor spheroid co-cultured with stellate cells in a 7-channel microfluidic plate	10
Intercellular interactions	3D culture	Liver cancer	Tumor cell co-cultured with stellate cells	8

**Table 2:** List of cancer related 3D printed microfluidic chip during the past five years

Research contents	3D printing method	Function of 3D printing	Notes	Ref.
Compared with the suitability of SLA and PloyJet method in printing microstructure of microfluidic device.	SLA and PolyJet bioprinting	Printed microstructure of microfluidic device	3D tumor spheroid; Liver cancer	178
Constructed a 3D microfluidic model to conduct the pharmacodynamic tests of an anti-CD147 monoclonal antibody.	Integrated printing	3D cell printing	3D culture; Liver cancer	39
Constructed a metastasis model on chip to investigate bone metastasis in breast cancer.	-	Fabricated the cast molds	3D culture; Breast cancer	26
Breast cancer cell morphology, migration, and the interaction with bone matrix on chip.	SLA	Constructed a 3D biomimetic bone matrix	3D culture; Breast cancer	179
Explored the effect of variable peptide-engineered exosomes in cancer immunotherapy.	-	Fabricated microfluidic culture chip	The yield and purity of engineered exosomes were improved, and the operation time was reduced.	180
A low-cost with ultralow detection limit immunoarray was developed to analyze the expression of multiple biomarker proteins in serum samples from cancer patients.	SLA	Fabricated microfluidic chip	Detected prostate cancer biomarker proteins in serum; Low sample volume.	181
Developed a “Lab-on-a-printer” and demonstrated its function by printed type Ⅰ collagen seeded with liver cancer cells.	Inkjet bioprinting	Fabricated chip and formed patterned biological structure by printing bio-ink	This platform integrated microfluidic mixer with inkjet dispenser on a chip.	182

**Table 3:** List of several representative CTCs isolation microfluidic chip in the past decades

Microfluidic technologies	Antigen-based selection	Antigen-independent selection	Basic properties	Advantages	Ref.	Application in cancer diagnosis
CTC-chip	√		The laminar flow of blood cells through the anti-EpCAM antibody-coated microposts in CTC-chip to capture CTCs.	Less damage to rare cells; Simplicity; Versatility; One-step manipulate.	183	184
The herringbone chip	√		The microvortices produced by herringbone grooves within the chip wall adequately mix the blood cells, increasing the interaction between CTCs and the anti-coated surface in chip.	Higher blood volume throughput; high capture efficiency and purity.	185	186-190
Geometrically enhanced differential immunocapture (GEDI)	√		The streamline deformation can help the target CTCs come into full contact with the immune coating on the wall; relative obstacle alignment uses the displacement generated by the impact between cells and obstacles to separate cells of different sizes.	High binding avidity and specificity; high cell capture efficiency and purity.	191	-
NanoVelcro Microfluidic Device	√					
CTC-ichip	√	√	The negative depletion of normal blood cells: using deterministic lateral displacement to isolate nucleated cells; using inertial focusing to align nucleated cells; deflecting and collecting magnetically tagged cells.	Automation; high-throughput; compatible with high-definition imaging and single-cell analysis.	60	192-194
Spiral chip		√	Spiral chip generates the inertial and Dean drag forces with continuous flow in curved channels to separate cells. The principle of separation is based on the physical difference between CTCs and blood constituents.	Stable streamlines distribution; high flow rates; ultra-high throughput; simplify the assistant procedures in clinical experiments; Less damage to CTCs.	195	196, 197
Straight chip		√	The straight chip take advantage of cells inertial migration in the straight microchannel to separate CTCs with high purity by manipulating flow rate ratio.	High purity collection; high recovery rate; high throughput; predictable and tunable cutoff size.	198	199
Nanotube-CTC-Chip		√	Carbon nanotube surfaces and microarray batch manufacturing is combined to capture and separate CTCs; Red blood cell lysis (RBCL) and preferential adherence can enrich CTCs.	High capture efficiency; high purity.	200	-
